# Molecular sequencing and morphological identification reveal similar patterns in native bee communities across public and private grasslands of eastern North Dakota

**DOI:** 10.1371/journal.pone.0227918

**Published:** 2020-01-23

**Authors:** Brian Darby, Russ Bryant, Abby Keller, Madison Jochim, Josephine Moe, Zoe Schreiner, Carrie Pratt, Ned H. Euliss, Mia Park, Rebecca Simmons, Clint Otto

**Affiliations:** 1 Department of Biology, University of North Dakota, Grand Forks, North Dakota, United States of America; 2 U.S. Geological Survey, Northern Prairie Wildlife Research Center, Jamestown, North Dakota, United States of America; 3 Humboldt State University, College of Natural Resources and Sciences, Arcata, North Dakota, United States of America; 4 Department of Biological Sciences, North Dakota State University, Fargo, North Dakota, United States of America; Sichuan University, CHINA

## Abstract

Bees play a key role in the functioning of human-modified and natural ecosystems by pollinating agricultural crops and wild plant communities. Global pollinator conservation efforts need large-scale and long-term monitoring to detect changes in species’ demographic patterns and shifts in bee community structure. The objective of this project was to test a molecular sequencing pipeline that would utilize a commonly used locus, produce accurate and precise identifications consistent with morphological identifications, and generate data that are both qualitative and quantitative. We applied this amplicon sequencing pipeline to native bee communities sampled across Conservation Reserve Program (CRP) lands and native grasslands in eastern North Dakota. We found the 28S LSU locus to be more capable of discriminating between species than the 18S SSU rRNA locus, and in some cases even resolved instances of cryptic species or morphologically ambiguous species complexes. Overall, we found the amplicon sequencing method to be a qualitatively accurate representation of the sampled bee community richness and species identity, especially when a well-curated database of known 28S LSU sequences is available. Both morphological identification and molecular sequencing revealed similar patterns in native bee community structure across CRP lands and native prairie. Additionally, a genetic algorithm approach to compute taxon-specific correction factors using a small subset of the most concordant samples demonstrated that a high level of quantitative accuracy could be possible if the specimens are fresh and processed soon after collection. Here we provide a first step to a molecular pipeline for identifying insect pollinator communities. This tool should prove useful for future national monitoring efforts as use of molecular tools becomes more affordable and as numbers of 28S LSU sequences for pollinator species increase in publicly-available databases.

## Introduction

### Importance of bees and need for widespread monitoring

Bees play a key role in the functioning of human-modified and natural ecosystems by pollinating agricultural crops and wild plant communities [1]. Nearly a quarter million flowering plant species, including many key crops, require insects and other animals to facilitate sexual reproduction [2]. The pollination services provided by bees to agriculture across the globe is valued at $181 billion USD, annually [3]. In the US, concern for wild and managed bees has grown in response to recent reports of decreasing species diversity and population trends [4–6]. Although emerging evidence does suggest a systematic decline in native bees, the US lacks a national monitoring program of native bee population trends and pollinator community composition across space and time. Lack of long-term monitoring to detect changes in species’ demographic patterns or shifts in bee community structure hampers pollinator conservation efforts globally [7]. The Pollinator Health Task Force [8] demonstrated the need for a national monitoring program for native bees, but development of such a program has been slow due to funding issues and numerous technical and logistical challenges.

The Pollinator Health Task Force [8] called for a concerted effort in improving taxonomic training and devoting resources to developing better genetic and taxonomic tools. One of the principle obstacles in developing a national monitoring program for native bees is the limited number of trained taxonomists needed to identify the approximately 4,000 species of bees native to the US. The lack of tools and taxonomists for identifying native bees presents an obstacle for a national monitoring program and creates a bottleneck for other native bee research projects across the country. This is especially true for research projects that employ passive sampling techniques, such as bee bowls [9] or Blue Vane traps, where the sheer number of bee samples collected do not permit lower-level taxonomic identification. Morphological identification of bees requires substantial resources and effort. Species determination depends on small morphological characters that require extensive entomological training and well-written dichotomous keys (e.g. [10]). Voucher specimens also need to be physically stored in archival conditions; insect collections are limited in space, personnel and funding [11]. Ecologists interested in conducting applied research on native bees often must rely on a limited number of trained taxonomists to identify all or a subset of specimens. Without extensive taxonomic expertise, researchers must then be resigned to conducting higher-level, community studies across different treatment or habitat types.

Molecular biology provides a promising, complimentary suite of techniques for identifying native bees and allowing new insights into bee demography, habitat preference, and foraging preferences (e.g., [12–14]). Genetic information can also be used to assess population structure, levels of gene flow, genetic diversity, and the occurrence of past events like bottlenecks and changes in connectivity [15]. The use of molecular techniques to identify bees has the potential to address the taxonomic bottleneck hampering ecological research and monitoring efforts. Here, we used native bees collected across grassland sites in the Prairie Pothole Region of North Dakota, USA, to create a genetic identification pipeline for North Dakota native bees. These methods are applicable to other ecosystems and questions involving pollinator diversity beyond the scope of this study.

Prior to European settlement, the Prairie Pothole Region was a mosaic of depressional wetlands with tall-grass prairie in the east to short-grass prairie in the west [16]. While limited research on native bee communities exists in this region, recent work suggests remnant patches of grasslands provide valuable habitat for native bees [17–19]. The Prairie Pothole Region is likely the most important region in the country for supporting managed honey bees [20]. Changes in land use, from grasslands to row crops, threaten pollinator forage and wildlife habitat [21–22]; these changes are exacerbated with reduced funding for land conservation programs.

Conservation areas in the Prairie Pothole Region exist under federal, state and private ownership. For example, the US Fish and Wildlife Service (USFWS) manages over 180,000 hectares of public land in North Dakota alone. The US Department of Agriculture (USDA) administers programs that incentivize landowners to implement conservation efforts on private lands. One such program, the Conservation Reserve Program (CRP) is the largest private lands conservation program in the US, with 9.2M and 0.52M ha enrolled nationally and in North Dakota, respectively [23]. The CRP targets marginal, environmentally sensitive, farmland by providing an annual rental payment if the landowner takes a portion of their land out of crop production to establish conservation cover. Although public and private conservation lands represent potentially important pollinator habitat, surprisingly little work has been done describing how bee communities vary across these areas. Understanding the different yet complementary roles private and public conservation areas play in supporting pollinators is important baseline information for natural resource managers.

The objectives of this project were to develop and test a molecular sequencing pipeline applied to native bee communities sampled in private and public grasslands in eastern North Dakota (S1 Appendix). The ideal molecular identification pipeline would a) target a commonly used locus (or loci) represented by many well-curated sequences in publicly available databases, b) produce accurate and precise identifications consistent with morphological identifications, and c) generate data that is both qualitative (detect correct number of species) and quantitative (detect correct proportional representation of species). To test this molecular pipeline, we assessed taxonomic concordance between traditional, morphological identification and molecular sequencing of >20,000 collected native bees. We also investigated the taxonomic resolution provided by each technique and discuss the strengths and limitations of the molecular pipeline. Finally, we used each technique to discern patterns in community structure of native bees collected on private lands enrolled in the CRP and public lands existing as National Wildlife Refuges. Our results provide a framework for using molecular techniques to rapidly identify native bee species in future research and monitoring efforts.

## Methods

### Sample collection

**Fig 1:**
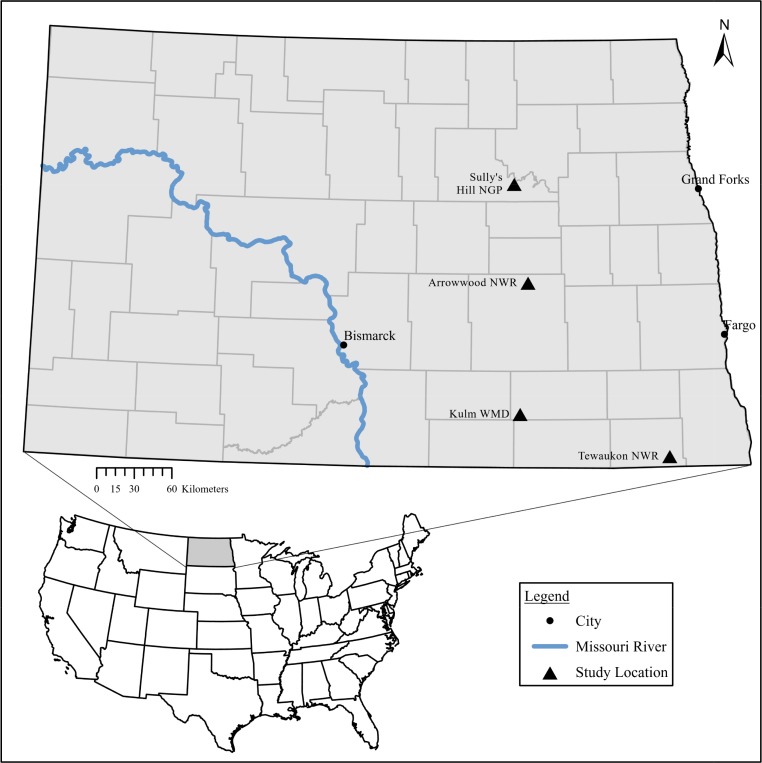
Study locations in eastern North Dakota. At each location two study sites were chosen, a native prairie grassland located on United States Fish and Wildlife Service managed lands and an adjacent restored prairie grassland enrolled in the United States Department of Agriculture’s Conservation Reserve Program.

Pollinator communities were sampled from four paired grasslands, herein referred to as “locations”, within the Prairie Pothole Region of eastern North Dakota in 2012 and 2013 (Fig 1). The four locations were centered around the following Fish and Wildlife Service lands: Arrowood National Wildlife Refuge (47.2693 N, -98.8522W), Tewauken Wetland Management District (45.9980 N, -97.3720 W), Kulm Wetland Management District (46.1475 N, -99.2999 W), and Sully's Hill National Game Reserve (47.9854 N, -98.9782 W). Each location consisted of 1) a native prairie grassland located on USFWS lands managed under the Native Prairie Adaptive Management (NPAM) system and 2) a restored grassland enrolled in the CRP under private ownership. Locations were approximately 64 ha and spaced 5 to 32 km apart. CRP grasslands included in this study consisted of Conservation Practice 1 (CP-1, Establishment of Permanent Introduced Grasses and Legumes), CP-2 (Establishment of Permanent Native Grasses), and CP-4D (Permanent Wildlife Habitat).

At each NPAM and CRP location, 20 transects were established at least 65 meters from each other, and 5 meters away from fences, wetlands, and shelterbelts. Vegetation data were collected along each transect but those data are not reported here. Transects were sampled approximately once every other week from May to September in 2012 and 2013, with a sample consisting of a single unscented Springstar blue vane trap [24] placed at one fixed end of the transect for 24 hours. Each trap was attached to a 5-foot rebar-post and hung by a 6-inch long tie wire so that each vane trap was at the approximate height of the nearby flora. Vane trap samples were placed in jars with labeled lids (containing date, site, and transect number) and were frozen at Northern Prairie Wildlife Research Center (NPWRC) after field work was completed each day. Queen bumblebees and threatened/endangered species (e.g. Dakota Skipper, *Hesperia dacotae*) observed in a trap were counted and released prior to removal from the location. We obtained approval to sample native bees on USFWS lands through a Research and Monitoring Special Use Permit with each USFWS refuge. We obtained verbal permission from private landowners for sampling CRP fields.

### Morphological identification

Pollinators were identified to the lowest possible taxonomic level at the NPWRC invertebrate lab in Jamestown, North Dakota. Established taxonomic keys [10] and online resources (e.g. Bug Guide, Discover Life) were used to identify specimens; a subset of specimens was verified by outside experts. After the identity was verified, specimens were archived at NPWRC and some are featured on the USGS Pollinator Library (https://www.npwrc.usgs.gov/pollinator/home). Although original identifications were made for each transect (i.e. each vane trap sample), the specimen identifications were pooled across transects within a location for the purposes of this study (and to conform to the molecular sequencing data).

### Molecular sequencing pipeline

#### Primer design

Molecular sequencing of whole bee pollinator communities by high-throughput amplicon sequencing requires primers that amplify short amplicons of highly variable regions, flanked by highly conserved regions. The variable regions are necessary to resolve species, while the conserved regions are necessary to ensure all targeted taxa are equally amplified with minimal amplification bias against any certain taxa. Ideal amplicons should be 500 to 550 bp in length, because the current MiSeq v3 chemistry allows 300 bp paired end reads. The maximum read length is then 600 bp, but because the quality of the last 50–75 nt is low, the ideal amplicon should leave at least 50–75 overlapping bases. Unfortunately, the loci that are most highly variable and also best represented in public databases, such as cytochrome oxidase I (COI; [25–26]), Elongation Factor 1-α (EF1-α; [27–28]), and *wingless* [28], did not contain, in our estimation, primer-binding sites that would be both conserved across all bee species and also result in an amplicon less than 550 bp. Instead, we collected and aligned 500 sequences from NCBI for both the 18S SSU and 28S LSU locus and identified one region in each gene: the V4-V5 region of the 18S small subunit (SSU, 555 bp in *Apis mellifera*), and the D2 region of the 28S large subunit (LSU, 507 bp in *Apis mellifera*). These gene regions met all three design requirements for this application:1) sequence conservation in the primer regions, 2) highly variable sequences between the primer regions, and 3) amplicons shorter than 600 bp (primers included).

#### Sample preparation

We sampled each specimen to account for community composition and for intraspecific genetic diversity. One mesothoracic leg from each specimen was placed into a 2.0 ml microcentrifuge tube representing its “community” (each date from each grassland field, with all transects pooled), and the other mesothoracic leg from each specimen was placed into a 2.0 ml microcentrifuge tube for representing its morphologically identified “taxa”. This resulted in 224 community tubes (consisting of a pool of one leg from each specimen from each of 2 grassland types x 4 locations x 28 sampling dates), and 56 taxa tubes (consisting of a pool of one leg from each specimen that had been morphologically identified to that taxa). In some cases, we combined the legs from two or more taxa tubes belonging to different families to minimize the total number of samples processed. We added five ceramic beads and two mealworm beetle (*Tenebrio molitar*) legs as inter-sample controls, and 500 ul genomic lysis buffer from the Qiagen 96-well Tissue and Cells kit to each tube. Tissue was lysed at room temperature overnight, then bashed for 10 min in a TissueLyser prior to proceeding with the recommended Qiagen 96-well Tissue and Cells kit instructions, which involved binding of DNA to silica gel, 2X washings of impurities, and finally elution of DNA into 60 μl elution buffer (EB), pH 8.0. This resulted in 190 DNA samples (160 community samples and 30 taxa samples) that were randomly arranged into two 96-well plates (with 90 samples and six blanks per plate).

#### Library preparation

First-round PCR was conducted in separate reactions for each loci using primers BD0150 (5’ TCGTCGGCAGCGTCAGATGTGTATAAGAGACA GTGCGGTTAAAAAGCTCGTAGTTG 3’) and BD0151 (5’ GTCTCGTGGGCTCGGAGATGTGTATAAGAGACA GAACCATACTTCCCCCGGAAC 3’) for 18S, and BD0152 (5’ TCGTCGGCAGCGTCAGATGTGTATAAGAGACA GTGAAACCGTTCAGGGGTAAACC 3’) and BD0153 (5’ GTCTCGTGGGCTCGGAGATGTGTATAAGAGACA GGTGTTTCAAGACGGGTCCTG 3’) for 28S. PCR reactions were conducted in 96-well plates (1x DreamTaq PCR buffer, 200 uM dNTPs, 0.2 uM each primer, 0.1 U DreamTaq polymerase, 2 ul DNA template) on Applied Biosystems Veriti thermocycler with an initial denaturation at 95°C (1 min), followed by 30 cycles of 95°C for 0:30, 55°C for 0:30, and 72°C for 0:30, followed by a final extension at 72°C for 5 minutes. For each plate, 10 ul of PCR product from the 18S amplification was pooled with 10 ul of PCR product from the 28S amplification and cleaned using the Zymo 96-well PCR Cleanup kit. We used 2 ul of the cleaned product as template for a second-round PCR amplification using dual-indexing primers with 8-nt Nextera barcodes (1x DreamTaq PCR buffer, 200 uM dNTPs, 0.1 uM each primer, 0.1 U DreamTaq polymerase, 2 ul DNA template) with an initial denaturation at 95°C (1 min), followed by 8 cycles of 95°C (30 s), 55°C (30 s), and 75°C (30 s), followed by a final extension at 72°C for 5 minutes. For each plate, 5 ul of second-round PCR product from each sample was pooled, cleaned using the Purelink PCR Cleanup kit (Invitrogen, Carlsbad, CA), and submitted for sequencing on the MiSeq Gene & Small Genome Sequencer (MiSeq Reagent Kit v3, 2x300 bp reads; Illumina, San Diego, CA), with each plate sequenced on a separate MiSeq run.

### Data analysis

#### Read processing

We obtained over 22 million quality-passed paired reads from each sequencing run. These paired reads were merged into one read, filtered to remove any reads with at least one predicted error [29], and dereplicated using USEARCH -fastq_mergepairs -fastq_filter -fastx_uniques commands [30–31]. For taxa samples, all sequences with, at least, 10 reads were aligned with MUSCLE [32] and examined visually in Geneious (Biomatters, Ltd., New Zealand) to identify true biological sequences apart from chimera and sequencing errors. Preliminary efforts suggested that various automated clustering and denoising algorithms came close to identifying true biological sequences, but not reliably enough for our purposes. For the community samples, all sequences with at least 2 reads were matched to the validated chimera-free biological sequences that were confirmed from the taxa samples using the “-otutab” algorithm of USEARCH [33] with a minimum 99% identity. The matches were tabulated with a custom Python/Biopython script [34] to reflect the total number of reads attributable to each taxon, for each sample.

#### Quantitation

An ideal molecular sequencing pipeline would not only be qualitative but also quantitative, yielding estimates of species counts in addition to species identities. In theory, the proportion of sequencing reads attributed to a particular species is proportional to the product of the number of individuals of that species, the number of haploid genomes in the tissue from each individual, and the number of rRNA gene copies per haploid genome for that species. In practice, though, the actual proportion of sequencing reads attributed to a species will also be affected by the completeness with which DNA is liberated from tissue (which could be affected by cuticle sclerotization), as well as a variety of PCR artifacts, such as the formation of chimeras (which should be more frequent if a species occurs with a closely related species), differential amplification efficiency, or differential binding affinity (e.g. if the primer-binding location is not conserved across all species). Nonetheless, even if one were to assume that the liberation of DNA from tissue was complete (or at least equivalent across taxa), and that PCR artefacts were also absent or equivalent across taxa, the rRNA gene copy number and the number of haploid genomes per specimen for each species, or a sort of “copy number correction factor”, has to be known in order to estimate proportional composition of specimens from just sequencing reads. To our knowledge, there is no analytical solution to this problem, nor is there a database of eukaryotic rRNA gene copy numbers, as there is for prokaryotes [35]. Darby et al. [36] applied a genetic algorithm approach to numerical optimization of rRNA gene copy number “correction factors” in soil nematodes. In short, the genetic algorithm is a rapid and highly parallelized “guess-and-test” algorithm that generates several thousand potential solutions of species-specific correction factors, each of which is used to determine what the theoretical sequence proportions would be based on the specimen counts and their respective copy number correction factor. The poor solutions (which have low concordance between expected and actual sequence proportions) are removed at each iteration, while the good solutions (which have high concordance between expected and actual sequence proportions) are duplicated and allowed to modify slightly, thus allowing the genetic algorithm to evolve at each iteration towards an optimal solution to correction factors that minimize the sums of squared errors between expected and actual data.

To determine if the molecular sequencing pipeline could be made quantitative, we applied the genetic algorithm from Darby et al. [36] to generate rRNA copy number correction factors to estimate the proportional representation of each species based on the proportional read counts. A key requirement of this approach is to have identical taxa represented in both the specimen-based dataset and the sequence-based dataset, so we identified 15 samples in which the genera identified by sequencing were perfectly concordant with the genera identified by morphology. We then ran the genetic algorithm for 10,000,000 iterations, using 1,000 genomes that all began with a correction factor estimation of 10 for each species. At each iteration, the “genomes” (correction factor estimates) were scored by computing sums of squared errors (between the morphologically identified specimen count and molecular sequence read count datasets), and the bottom third poorest genomes were removed while the top third were duplicated and allowed to mutate by an amount drawn from a normal random distribution with a standard deviation of 5.

#### Community composition

Bee pollinator abundance and richness were modeled with a Generalized Linear Mixed Model to test the effects of location, management type (CRP vs NPAM), and location*management type interaction on the response variables abundance (of specimens), morphological richness (as determined by morphological identification of specimens), and molecular richness (as determined by molecular sequencing of 28S LSU). All three response variables were assumed to be drawn from a Negative Binomial distribution (modeled with a log-link). In the case of abundance, each individual vane trap was used as the error term (modeled as repeated measures with first-order auto-regressive R-side residuals). In the case of both richness variables, the vane traps were pooled within a sampling date so that the sampling date was the error term.

Sample-based species accumulation curves were calculated with iNEXT [37–38], using presence-absence data from both the morphological specimen identifications and the 28S LSU sequencing data, to determine if grassland types differed in their total predicted number of species. Principal components analysis (PCA) was computed with the *vegan* package of R [39] to determine if the multivariate composition of species differed between grassland types.

## Results

### Species identifications

Of the 103 morphologically identified taxa, 75 were identified to a single species, and 28 were identified to the level of genus or a species complex. From these morphologically identified specimens, we obtained 87 unique molecular sequences from the 28S LSU locus, and 45 unique molecular sequences from the 18S SSU locus. The 28S LSU sequences were more capable of discriminating between taxa than the 18S SSU sequences, as the median difference from the nearest validated sequence for the 28S locus was 9 nucleotides in this community, but most unique sequences from the 18S locus were within just 1 nucleotide of the next most similar validated 18S sequences.

There were only ten instances of a 1:1 link between a single morphologically identified taxa and a single 28S LSU molecular sequence. More commonly, there were either multiple morphological taxa linked to a single molecular sequence, multiple molecular sequences linked to a single morphological taxon, or both (S2 Appendix). In some cases, the 28S locus was able to resolve more species than morphological identifications. For example, morphology was unable to resolve specimens of the genus “*Nomada*”, but 28S amplicons yielded four different sequences, each a minimum of 22 nucleotides from each other. Similarly, 28S amplicons resolved four different sequences of “*Andrena*” that were each at least 3 nucleotides different from each other that were not resolved by initial morphological identifications. However, in some cases, sequencing of the 28S locus resulted in fewer species than morphological identifications, often due in part to the ambiguity of the morphological identifications. For example, while morphology grouped specimens of the genus *Ceratina* into three groups (“*C*. *calcarata”*, “*C*. *dupla”*, or “*C*. *dupla/calcarata”*), 28S amplicons only yielded two sequences (API213 and API243), which differed by 3 nucleotides. Similarly, while morphology grouped specimens of the genera *Eucera* and *Svastra* into four taxa (*Eucera (Synhalonia)*, *Eucera* NA, *Svastra obliqua*, and *Svastra* NA), 28S amplicons only yielded two sequences (API217 and API235) which differed by 9 nucleotides.

### Species richness

Of the 18,876,520 reads from merged community samples (n = 124), 1,642,701 passed all filtering and quality checks and were successfully mapped to a known 28S bee sequence (resulting in a median of 12,667 LSU reads per community sample), while 1,834,381 quality-passed reads were mapped to the beetle 28S calibration sequence. Similarly, 965,281 quality-passed reads were mapped to a known 18S bee sequence (resulting in a median of 6857 SSU reads per community sample), while 69,248 quality-passed reads were mapped to the beetle 18S calibration sequence. The remaining reads were discarded as either singletons, chimeric, or non-bee sequences (the 18S locus was conserved enough to amplify plant DNA).

**Fig 2:**
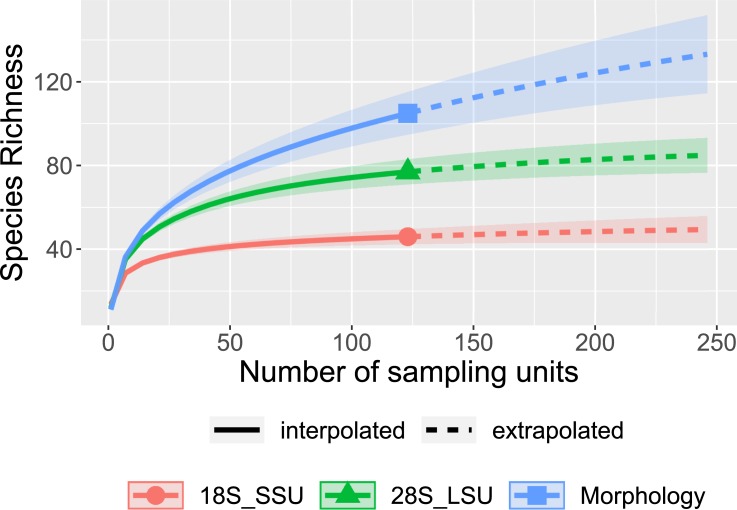
Comparison of species discovery rates. Rarefaction curves showing the expected number of species (vertical axis) relative to the number of sampling units (horizontal axis), for the 18S SSU locus, 28S LSU locus, and the morphological specimen identifications.

**Fig 3:**
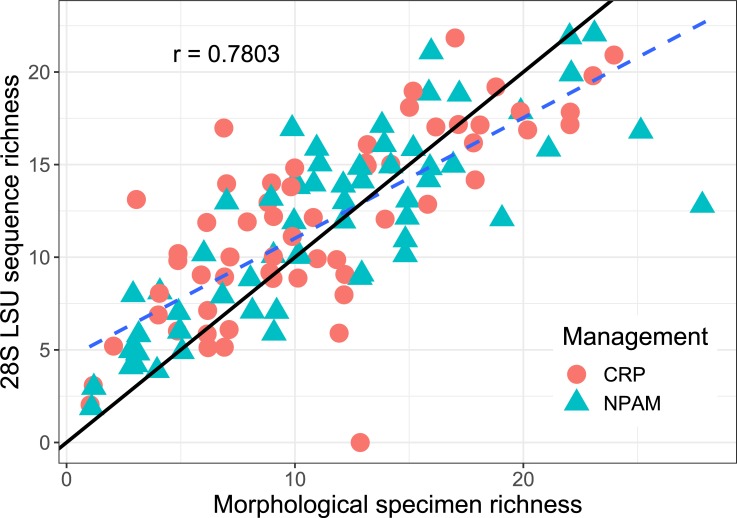
Morphological and molecular methods comparison. Correlation between species richness as detected by molecular sequencing (vertical axis) relative to species richness as detected by morphological specimen identification (horizontal axis), for both Conservation Reserve Program (CRP, circles) and Native Prairie Adaptive Management (NPAM, triangles) management regimes. Each marker represents a sample, the solid line represents the 1:1 reference line, and the dashed line represents the empirical best fit (r = 0.7803, *p* < 0.0001).

Species rarefaction curves for both molecular loci (28S LSU and 18S SSU) appear to approach 124 samples; additional sampling from within this model would likely discover few additional species (Fig 2). While it appears that morphological identification yielded more taxa (103) than 28S LSU sequencing (87), many of the morphological taxa were likely inflated by the inherent difficulties of morphological identifications across a large multi-year project. For example, after observing several thousand specimens it is possible to observe new characters over time such that a specimen that might have been identified to one taxon at the beginning might be identified as a different, or perhaps more specific, taxon at the end of the project. The taxa identified by 28S LSU sequencing, however, are likely to be more consistent and reliable across the entire sampling effort because the nucleotide sequences do not change within the span of a project. The molecular species richness that resulted from these 124 community samples was positively correlated with the species richness determined morphologically (Fig 3, r = 0.7803, p < 0.0001). We believe that tissue degradation was likely the main reason for these instances of failed PCR amplification (see Discussion).

### Quantification

**Fig 4:**
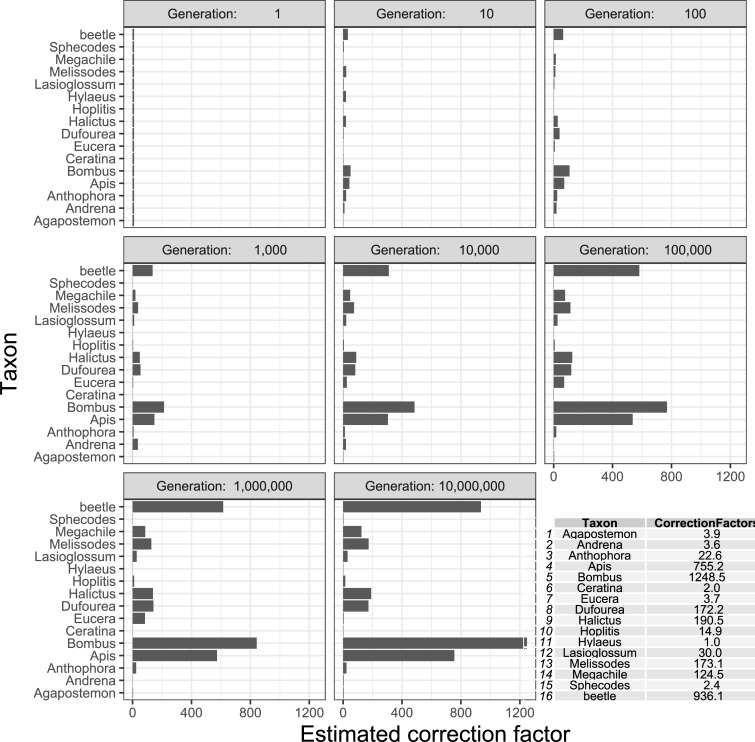
Results of correction factor genetic algorithm. The top copy number correction factor estimates (horizontal axis) is plotted for each taxon (vertical axis) after eight logarithmically spaced generations of the genetic algorithm (panels). Inset: tabular summary of the final correction factor estimates for each taxon.

**Fig 5:**
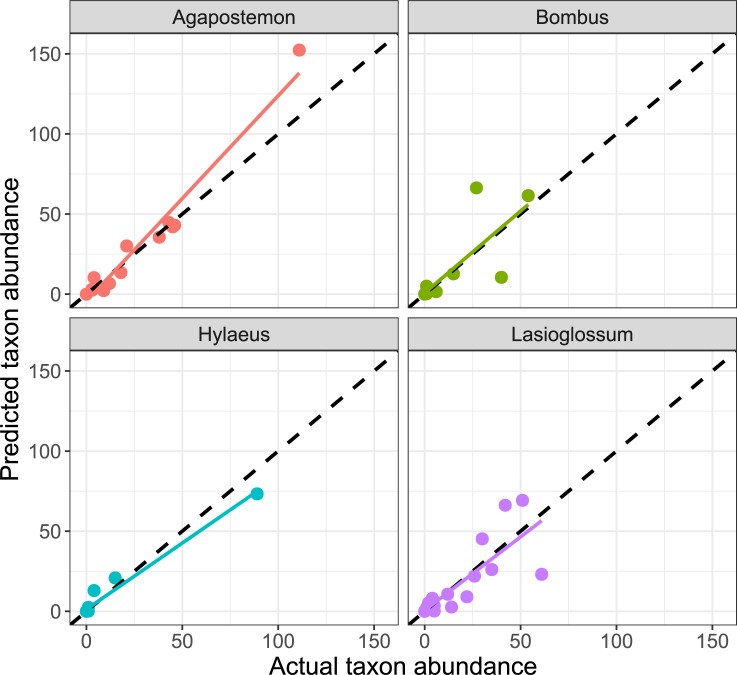
Quantifying abundance from read counts. Copy number correction factors from the genetic algorithm (Fig 5) were used to retroactively estimate the abundance (vertical axis) of 15 bee taxa (top four most frequent taxa shown here) relative to their actual specimen counts (horizontal axis). Each marker color represents a different taxon, the dashed black line represents the (ideal) 1:1 reference line; each colored line represents the best fit for that taxon.

Because of discrepancies between the molecular and morphological taxa, it was not possible to attempt rRNA gene copy number correction factors for all taxa from all samples. Instead, we selected 15 samples in which the genera for the morphological identifications were completely congruent with the genera from the molecular samples (which happened to be 15 bee genera). We then estimated rRNA gene copy number correction factors for these 15 bee genera (plus the beetle calibrator) using the genetic algorithm optimization proposed by Darby et al. [36]. After 10,000,000 generations, the genetic algorithm eventually converged upon relative correction factors that ranged from as low as 1x (*Hylaeus*) to as high as 1,248x (*Bombus*) (Fig 4 inset). These are not necessarily rRNA gene copy numbers per haploid genome, but rather a generalized correction factor that incorporates both rRNA copy number per haploid genome, the number of haploid genomes per cell, and the number of cells in the leg tissue that was collected from each specimen. For example, the correction factor for *Lassioglossum* was 30.0, which is about twice the correction factor for *Hoplitis* (14.9), implying that one leg of *Lassioglossum* will tend to yield approximately twice the number of amplicons as a leg from *Hoplitis*, and will therefore be disproportionately overrepresented in a sample by about twice as much as *Hoplitis* (but only by one-quarter as much as *Megachile*, which has a correction factor of 124.5). These relative correction factors were then retro-actively applied to the sequence read count data to estimate the predicted number of specimens from each genus. The overall correlation of predicted to actual abundance of all genera from the 15 selected samples was 0.8859 (95% CI: 0.8552 to 0.9104), and this correlation was strongest in the four most frequent taxa: *Agapostemon*, *Bombus*, *Hylaeus*, and *Lasioglossum* (Fig 5).

### Community composition

**Fig 6:**
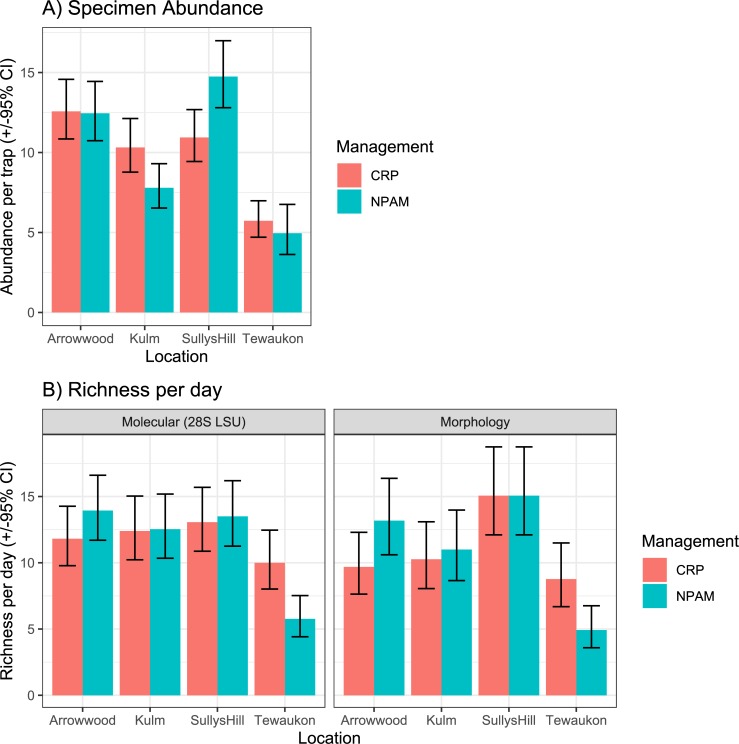
Bee abundance and richness. A) Mean (± 95% CI) number of individual bees collected in vane traps (vertical axis) across all sampling points for both years (2012 and 2013), at four locations (Arrowwood, Kulm, Sully’s Hill, and Tewaukon), for both Conservation Reserve Program (CRP) and Native Prairie Adaptive Management grasslands (NPAM). B) Mean (± 95% CI) richness of bees per sampling day (all vane traps pooled together) obtained by molecular sequencing (28S LSU) and morphology, at four locations (Arrowwood, Kulm, Sully’s Hill, and Tewaukon), for both Conservation Reserve Program (CRP) and Native Prairie Adaptive Management (NPAM).

**Fig 7:**
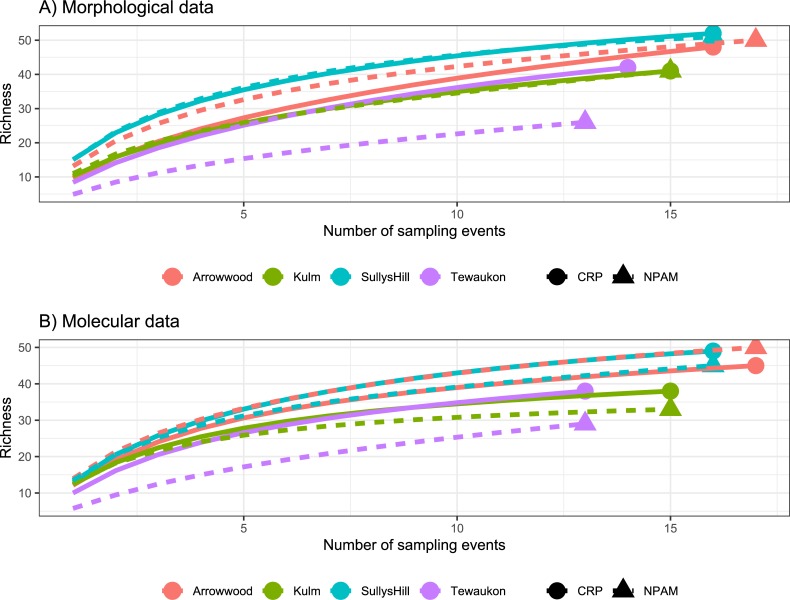
Species rarefaction curves. Rarefaction curves showing the expected number of species relative to the number of sampling units (horizontal axis), for the four locations (red = Arrowwood, green = Kulm, blue = Sully’s Hill, purple = Tewaukon) and two management regimes (dashed lines = CRP, solid lines = NPAM) based on A) morphological specimen identification, or B) molecular 28S LSU sequencing.

**Fig 8:**
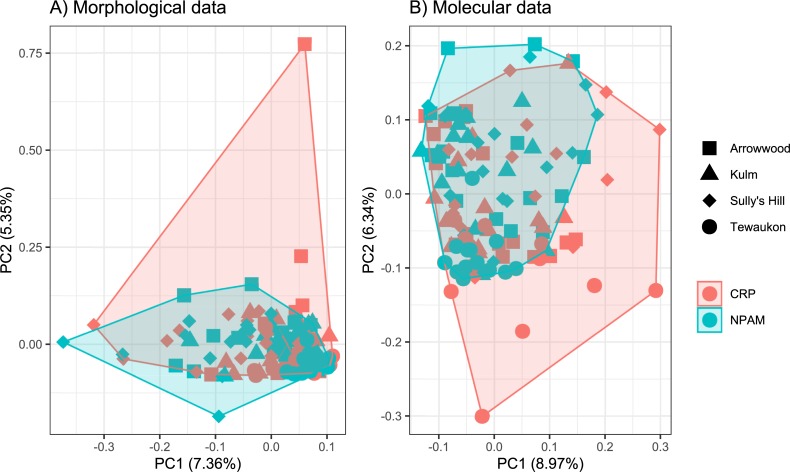
Ordination of Principal Components Analysis. Bee samples from Conservation Reserve Program (CRP) and Native Prairie Adaptive Management (NPAM) grassland types across four locations (Arrowwood, Kulm, Sully’s Hill, Tewaukon) plotted along principal component 2 (vertical axis) and principal component 1 (horizontal axis) based on A) morphological specimen identification, or B) molecular 28S LSU sequencing. The shaded region represents the minimum convex polygon encompassing all samples and all locations for a given management regime.

Trends of bee abundance (recovered in the vane traps) varied by location, and the interaction of location*management type, but not by management type as a main effect. (Table 1, Fig 6). Similarly, species richness per sampling date varied by location, and the interaction of location* management type, but not by management type as a main effect, and this was true for both the morphological identifications and the molecular sequences (Table 1, Fig 6). Species accumulation curves were comparable for both the specimen-based data and the sequence-based data as both showed that, for the number of samples collected, the Arrowwood and Sully’s Hill locations had greater species richness than Kulm or Tewaukon locations, regardless of whether the management was CRP or NPAM (Fig 7). Similarly, Principal Components Analysis were comparable for both specimen-based and sequence-based data as both showed that the multivariate composition of samples was more distinct between sampling locations, and largely overlapping (or indistinct) between management types (Fig 8).

**Table 1:** ANOVA results for bee specimen abundance.

EFFECT	DF (num)	ABUNDANCE	SPEC_RICHNESS	SEQ_RICHNESS
LOCATION	3	28.05***	13.50***	13.38***
MANAGEMENT	1	0.26	0.32	1.40
LOCATION*MANAGEMENT	3	4.74**	3.76*	3.83*

*p<0.05

**p<0.001

***p<0.0001.

ABUNDANCE indicates number of bee individuals per vane trap as has 155 denominator degrees of freedom.

SPEC_RICHNESS indicates species richness per day as determined by morphological identification of specimens. SEQ_RICHNESS indicates species richness per day as determined by molecular identification of sequences. Both versions of richness have 13 denominator degrees of freedom.

## Discussion

Research and monitoring of native bee communities is constrained by a limited number of trained taxonomists and alternative tools for identifying native bees [40]. The objective of this project was to test a molecular sequencing pipeline much like the same amplicon sequencing that is used to characterize microbial communities [41], fungi [42], protists [43], nematodes [36], and bee-collected pollen [44]. Our first goal was to target a commonly used locus that already has many well-curated sequences in publicly available databases. Although we were not able to accommodate the most commonly used loci (such as COI or EF1-α) we did find success with the 28S LSU rRNA locus, which has a growing number of identified sequences in the publicly-available databases such as NCBI and SILVA [45]. Our second goal was to produce accurate and precise identifications that are consistent with morphological identifications. We found the 28S LSU locus to be very capable of discriminating between species, and in some cases even resolved instances of cryptic species or ambiguous species complexes. Our final goal was to generate data that were both qualitative (able to resolve the correct number of species) and quantitative (able to detect the correct proportional representation of species). Here, the amplicon sequencing method produced data that were reasonably qualitative (in that the sequence-based species richness was positively correlated with the specimen-based species richness), but unfortunately were not quantitatively accurate. The inability to accurately quantify the number of individuals in a mixed sample was likely due to degradation of tissue and nucleic acids prior to PCR amplification. However, a procedure to compute taxon-specific correction factors using a small subset of the most concordant samples demonstrated that a high level of quantitative accuracy could be possible if the specimens are fresh and processed soon after collection. Overall, our results highlight the potential for molecular sequencing to characterize bee community composition in a scalable, high-throughput, and reproducible procedure. Thus, research and monitoring efforts designed to track changes in bee presence/absence or bee richness across space and time may find molecular sequencing an effective tool for rapidly processing bee samples; further refinements will increase the reliability of these approaches to track changes in species abundance across space and time.

### Species identifications

A molecular identification approach has much to offer community-level studies in terms of objectivity and repeatability. Identifying specimens to reconstruct community composition for a large number of samples is a different challenge than identification of specimens for alpha taxonomy or phylogeny reconstruction. In the latter, the researcher typically has developed a deep familiarity with one or a few taxa and is less familiar with other, distantly related taxa. In this case, it is often necessary for the researcher to spend considerably more time and effort on these specimens to discover new species or to resolve unfamiliar species complexes. However, in the case of identifying specimens from a community composition project, the operator may be discouraged from spending too much time on identifying any one specimen, especially if it appears to be rare and not a large proportion of the community. This is why many community composition identification projects have two undesirable characteristics: 1) several ambiguous taxa that represent specimens which were not, or could not have been, identified to the same level of certainty as other specimens (often colloquially referred to as a “junk” genus), and 2) the taxonomic identification of certain species can sometimes change throughout the course of a project, often as the operator(s) gain more familiarity with the specimens or observe additional characters that were always present but not previously observed. Perhaps the strongest argument in favor of identifying specimens for community composition by sequencing is that both problems are nearly eliminated. As long as the chosen locus varies enough to resolve specimens at the desired taxonomic level, then their molecular sequence is accurate, precise, and standardized throughout the likely time span for such a project.

We found the 28S LSU locus to be successful at resolving species identity, and was more discriminating than the 18S SSU locus, which would only be capable of resolving genus or family. The 18S SSU locus was highly conserved at its primer binding sites, even amplifying plant rRNA; use of this locus could be helpful at a different taxonomic scale, allowing identification of insects at a broader level. The main limitation of the 28S LSU locus is that there is not a large database of complete and well-identified reference sequences for most geographic locations. Although there is a growing availability of sequences in NCBI and SILVA [45], we did not find any of the native North Dakota bee species in the SILVA database. Thus, a long-term monitoring project that uses the 28S LSU locus for high-throughput amplicon sequencing may need to supplement the available databases with an effort to sequence a subset of morphologically identified specimens.

### Species richness

Our study shows molecular sequencing allows for an accurate estimation of species richness even if a complete reference database is not available to obtain species identification. Depending on a monitoring effort’s objectives, estimating species richness alone, in the absence of species identification, may suffice. In our project, estimates of species richness were positively correlated between molecular and morphological methods (r = 0.7803, Fig 3). Some of the discrepancy between molecular and morphological species richness was due to imprecision in the morphological identifications, such as clusters of multiple morphological taxa (e.g. the three morphological groups “*Ceratina calcarata”*, “*Ceratina dupla”*, and “*Ceratina dupla/calcarata”* actually yielding just two molecular sequences: “*Ceratina calcarata”*, and “*Ceratina dupla*”). The rest of the discrepancy between molecular and morphological species richness appeared due to a lack of DNA leading to failed PCR amplification of certain specimens (likely due to degradation of DNA in the tissue of the specimens). In one sample, there were zero molecular sequences recovered, despite 13 morphological species in the original sample (Fig 3). Again, this was likely a consequence of either degraded tissue in all of the specimens, or an idiosyncratic failure of either the gDNA extraction, purification, or PCR amplification. The specimens were sampled in 2012 and 2013, identified morphologically, and then stored frozen at -20°C until they were processed for molecular sequencing in 2017. The number of reads from the beetle legs (which were sampled fresh right before processing for DNA extraction) far outnumbered the reads for bees. We do not believe that the beetle legs were much larger than most bee legs, nor had obviously more cells or were much easier to disrupt. Instead, we believe that the bee samples must have experienced some degradation of tissue and nucleic acids between the time they were first sampled to when they were processed for DNA extraction. Thus, we recommend that future sampling efforts and monitoring projects use fresh tissue from recently sampled specimens if the goal is to characterize multiple specimens in a community using molecular amplicon sequencing.

### Quantification

The ideal high-throughput molecular sequencing protocol would be both qualitative (accurate identification of specimens, and positive correlation with overall community species richness) and quantitative (accurately reflect the proportional representation of the species within a community). Relatively accurate quantification of species abundance was possible by applying taxa-specific correction factors to the amplicon sequencing method of molecular identification. Without correction factors, this method was not capable of estimating relative abundance of each species in the community for the following reasons: 1) lack of 1:1 concordance between molecular and morphological species identifications, 2) failed representation of some species due to degraded tissue (as discussed in “Species Richness”), and 3) most importantly the nature of the multi-copy rRNA gene being used. The rRNA operon (which contains both the 18S SSU and the 28S LSU loci) occurs in multiple tandem repeats and varies considerably in copy number between species. For example, rRNA gene copy number varies from 45 to 1023 just in mosquitos [46]. While it is true that rRNA copy number is phylogenetically autocorrelated (closely related species tend to have a relatively similar rRNA copy number), there is variation within a genus and potentially even within a species. For a high-throughput molecular identification pipeline to quantify species relative abundance, correction factors would need to be developed for each taxon in the community. These correction factors would account for both the varied number of rRNA gene copies in a haploid genome, as well as the number of cells in the portion of tissue (e.g. leg) from which gDNA is extracted.

We computed such correction factors by selecting the 15 samples that were most concordant between the molecular and morphological identifications, and applied a genetic algorithm optimization procedure proposed by Darby et al. [36] to generate correction factors for soil nematodes in a similar circumstance. This approach resulted in correction factors that successfully reconstructed the relative abundance of the four most frequently occurring taxa. However, the correction factors were mediocre to poor at estimating the relative abundance of the less frequently occurring taxa. Nonetheless, these data show promise for using amplicon sequencing to generate quantitative data on species relative abundance, but only under relatively stringent criteria. First, the tissue that is used for gDNA extraction must be of high quality and without degradation of available DNA. We recommend processing samples as soon as possible, and with minimal storage even in freezing condition. Secondly, there must be a high level of concordance between the molecular sequencing data and the morphologically identified specimen counts that are used as the reference training data for the genetic algorithm. One way to accomplish this might be to perform the molecular sequencing and identification first, then revisit the specimens for morphological identification and counting after a species list is generated using molecular sequencing. The morphological counts could then be used as the quantitative data itself or used as training data for the genetic algorithm-based estimates of correction factors to be applied to other samples. Even without quantitation, monitoring programs in the US have demonstrated the effectiveness in using species occurrence data to infer large-scale shifts in the distributions of vertebrate wildlife [47–49]. Although tracking changes in species abundance is often the preferred standard for biological monitoring programs, species occupancy can be an effective surrogate [50].

### Community composition

The Pollinator Health Task Force [8] called for a stronger research focus on evaluating the role of public and private lands in supporting pollinator health. Here we showed native bee community structure varied more between sampling locations (Arrowwood, Kulm, Sully’s Hill, and Tewaukon) than they did between grassland management types (Conservation Reserve Program vs. Native Prairie Adaptive Management). This is significant given that recent declines in native bee abundance have been attributed in-part to the conversion of grasslands such as CRP to row crop [6]. Our results suggest that marginal farmland that is enrolled in the CRP has the potential to become viable native bee habitat, at levels similar to native prairie.

Our data showed the relationship between bee abundance and richness to grassland type was conditional on the location where bees were trapped. The location*management interaction we observed could be due to differences in location-specific management of the grasslands we sampled, or due to local landscape effects. The native bees we observed have a variety of life-history traits, but in general native bees require nesting habitat and floral resources to complete their lifecycle. Indeed, native bee diversity and network structure is higher in areas with increased habitat and floral resource heterogeneity [51–52, 17]. Quantifying bee response to floral resource availability across grassland types was beyond the scope of our current research but will be the focus of a subsequent investigation. Here, we were successful in showing that molecular sequencing yields spatial patterns in grassland bee communities similar to the patterns detected using traditional morphological identifications.

## Conclusion and proposed workflow

The objective of this project was to test a molecular sequencing pipeline to see if it could be used as part of a long-term sampling or monitoring effort. We found the amplicon sequencing method to be a qualitatively accurate representation of the sampled bee community richness and species identity, especially when a well-curated database of known 28S sequences is available. Finally, we described viable steps to improve quantitation by applying correction factors and using fresh material.

To incorporate this molecular sequencing pipeline into a large-scale or long-term monitoring program, we recommend the following steps based on our experiences from this project: 1) collect samples using either a passive or active technique, such that the total number of individuals per sample is small enough so that one leg from each sample fits within a 2-ml centrifuge tube, 2) pull one mesothoracic leg from each specimen and pool within a sample into a single 2-ml centrifuge tube, if possible, 3) extract gDNA using an overnight tissue lysis followed by a bead-bashing protocol, and avoid freezing tissue for extended periods, 4) amplify 28S LSU locus by PCR and prepare barcoded libraries for 2x300 bp sequencing, 5) after sequencing, merge, filter, and map reads to reference database, or to de novo OTUs, 6) using list of species or OTUs from each sample, count specimens and verify sequencing data with morphological identifications, and 7) use the specimen counts as quantitative data, or as a training dataset to estimate read count correction factors if needed.

## Supporting information

S1 Appendix Conceptual diagram of sequencing workflows. (PPTX)

S2 Appendix Concordance between morphological and molecular identifications. (PPTX)
