# Supplementary material for: Molecular sequencing and morphological identification reveal similar patterns in native bee communities across public and private grasslands of eastern North Dakota
Source: PLoS One. 2020 Jan 23;15(1):e0227918. doi: 10.1371/journal.pone.0227918 (PMC6977755; doi:10.1371/journal.pone.0227918)
Supplement: S1 Appendix — (PPTX) [file pone.0227918.s001.pptx]

## Slide 1
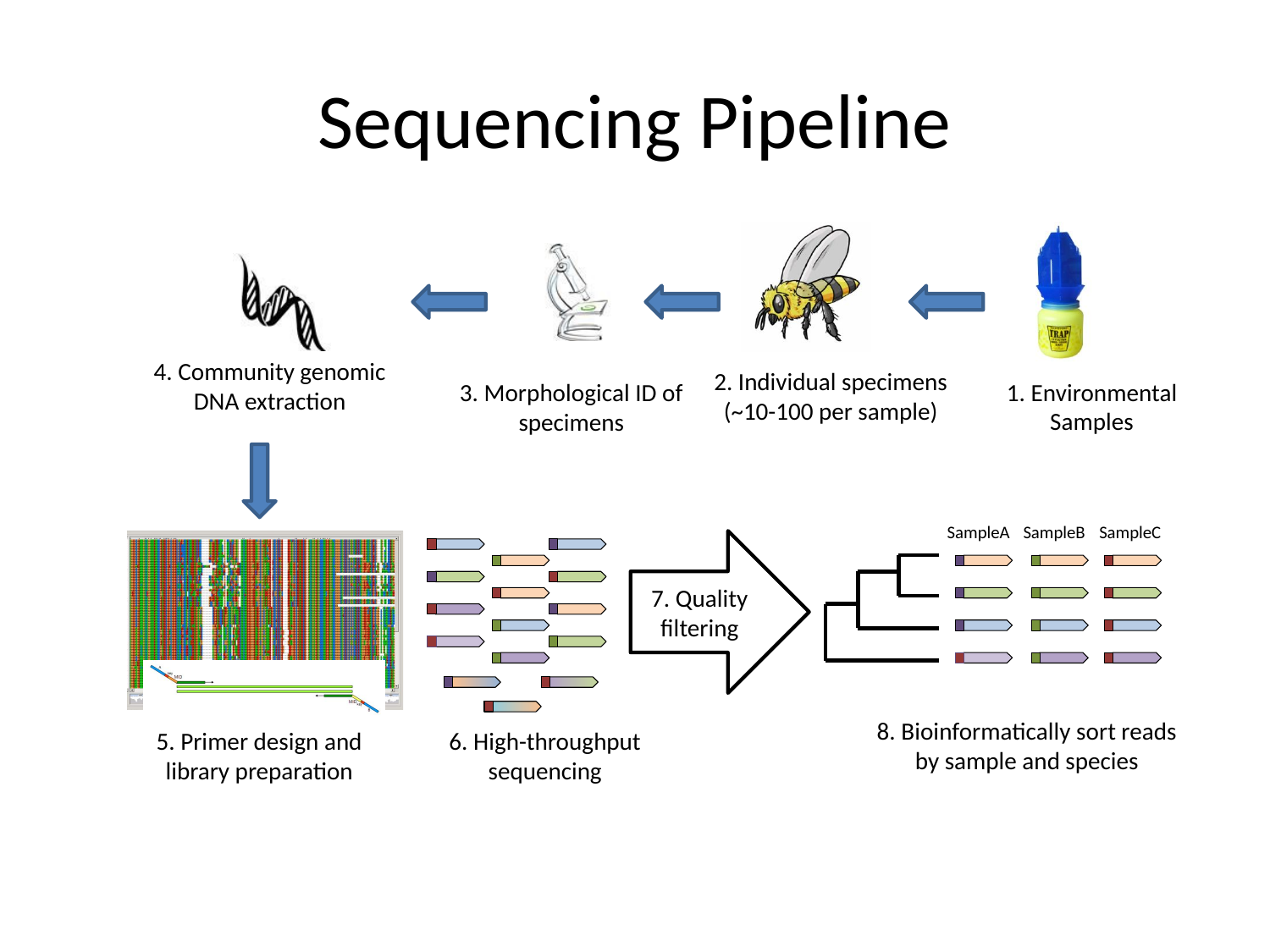

# Sequencing Pipeline
4. Community genomic DNA extraction
2. Individual specimens (~10-100 per sample)
3. Morphological ID of specimens
1. Environmental Samples
SampleA
SampleB
SampleC
7. Quality filtering
8. Bioinformatically sort reads by sample and species
5. Primer design and library preparation
6. High-throughput sequencing

## Slide 2
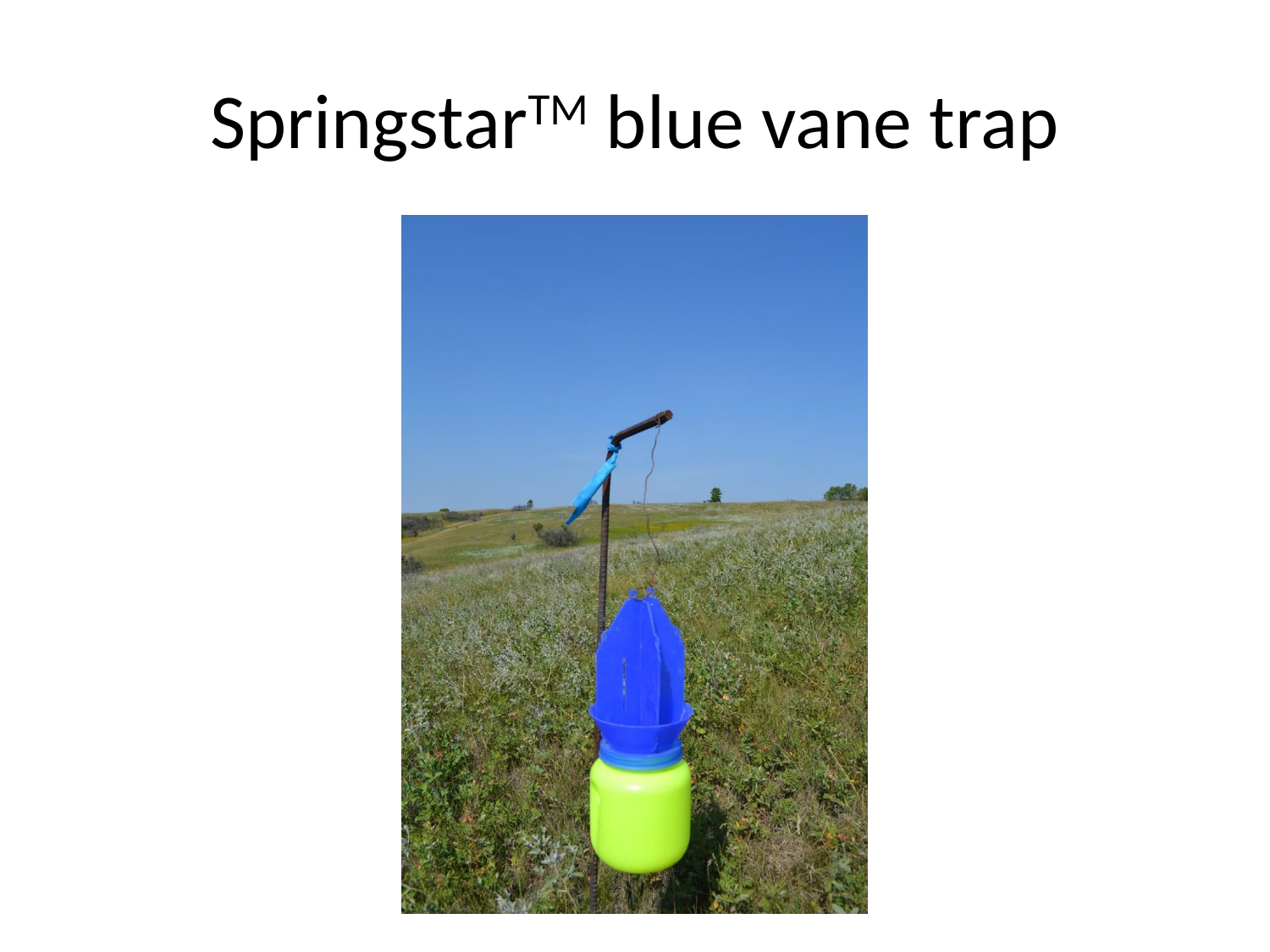

# SpringstarTM blue vane trap

## Slide 3
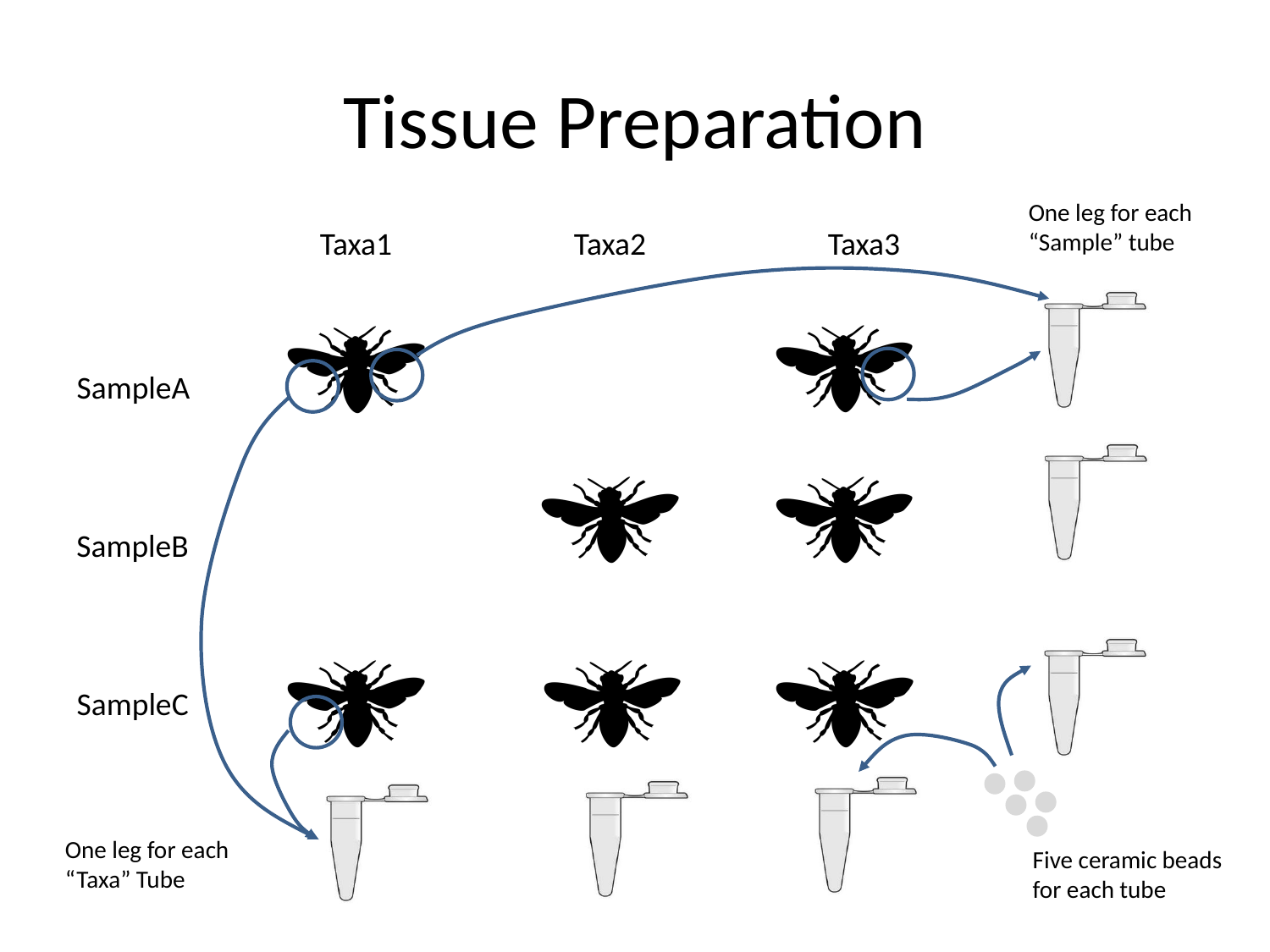

# Tissue Preparation
One leg for each “Sample” tube
Taxa1
Taxa2
Taxa3
SampleA
SampleB
SampleC
One leg for each “Taxa” Tube
Five ceramic beads for each tube

## Slide 4
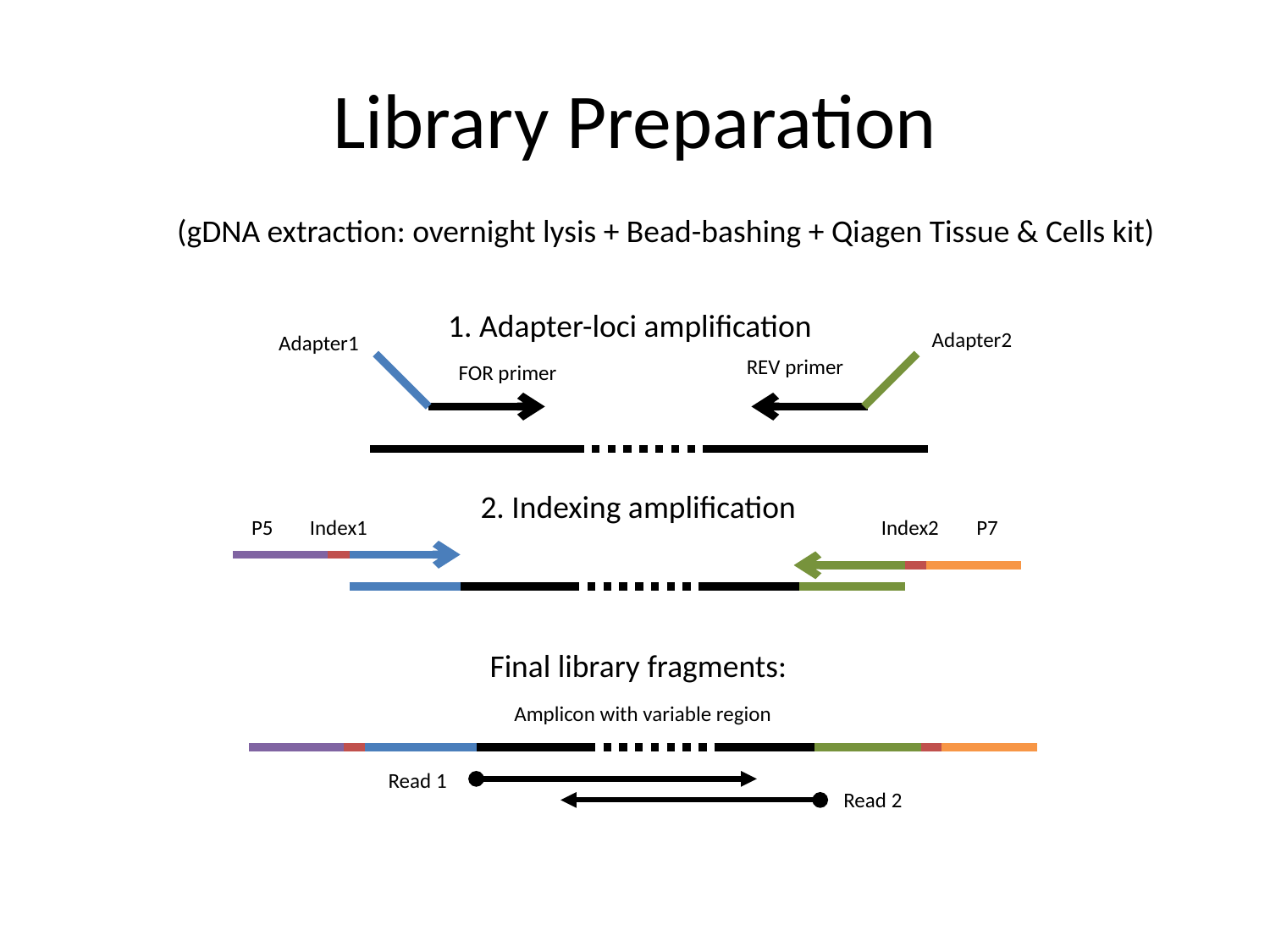

# Library Preparation
(gDNA extraction: overnight lysis + Bead-bashing + Qiagen Tissue & Cells kit)
1. Adapter-loci amplification
Adapter2
Adapter1
REV primer
FOR primer
2. Indexing amplification
P5
Index1
Index2
P7
Final library fragments:
Amplicon with variable region
Read 1
Read 2
