# Supplementary material for: Molecular sequencing and morphological identification reveal similar patterns in native bee communities across public and private grasslands of eastern North Dakota
Source: PLoS One. 2020 Jan 23;15(1):e0227918. doi: 10.1371/journal.pone.0227918 (PMC6977755; doi:10.1371/journal.pone.0227918)
Supplement: S2 Appendix — (PPTX) [file pone.0227918.s002.pptx]

## Slide 1
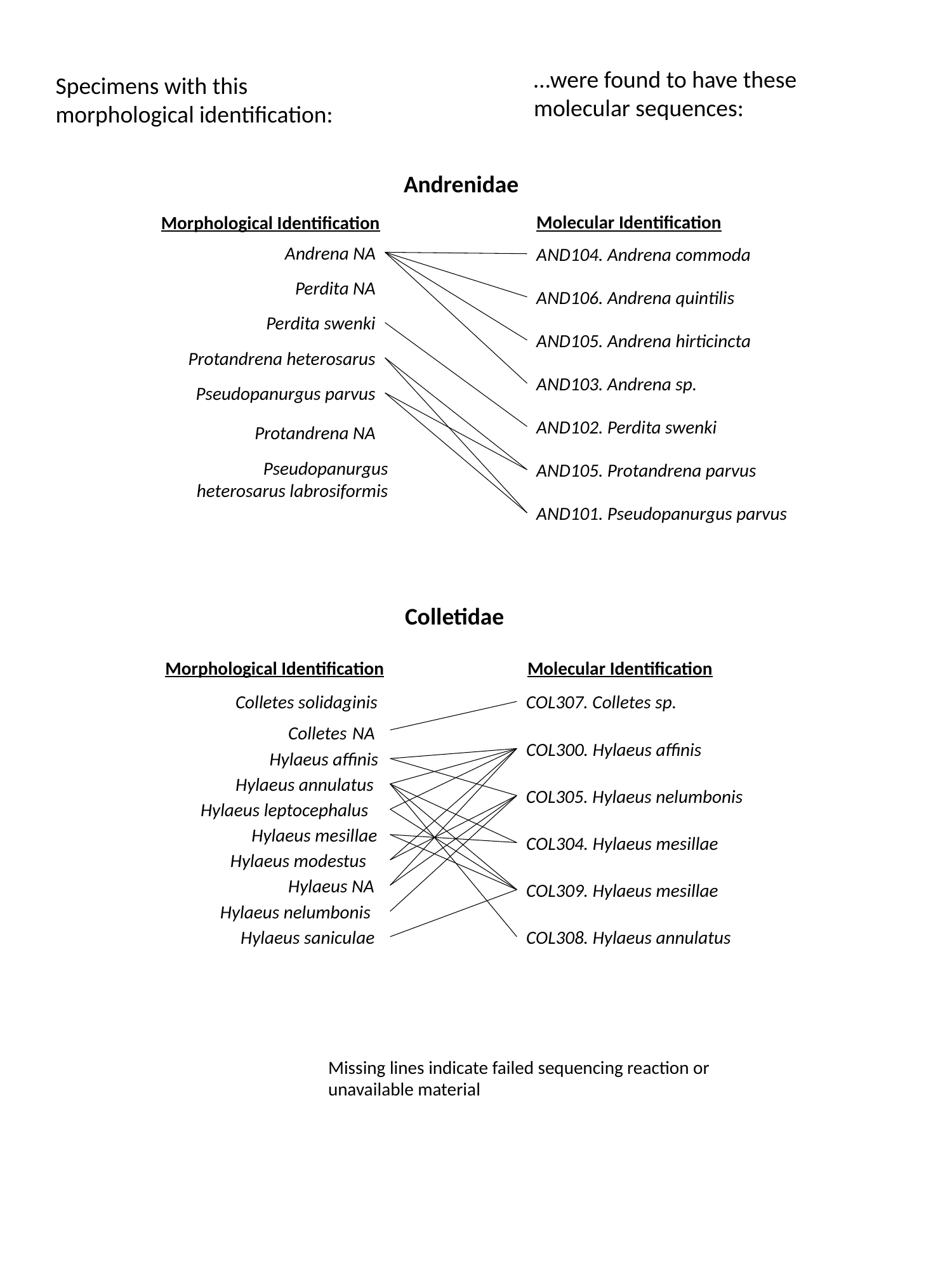

…were found to have these molecular sequences:
Specimens with this morphological identification:
Andrenidae
Molecular Identification
Morphological Identification
Andrena NA
AND104. Andrena commoda
Perdita NA
AND106. Andrena quintilis
Perdita swenki
AND105. Andrena hirticincta
Protandrena heterosarus
AND103. Andrena sp.
Pseudopanurgus parvus
AND102. Perdita swenki
Protandrena NA
Pseudopanurgus heterosarus labrosiformis
AND105. Protandrena parvus
AND101. Pseudopanurgus parvus
Colletidae
Morphological Identification
Molecular Identification
Colletes solidaginis
COL307. Colletes sp.
Colletes NA
COL300. Hylaeus affinis
Hylaeus affinis
Hylaeus annulatus
COL305. Hylaeus nelumbonis
Hylaeus leptocephalus
Hylaeus mesillae
COL304. Hylaeus mesillae
Hylaeus modestus
Hylaeus NA
COL309. Hylaeus mesillae
Hylaeus nelumbonis
Hylaeus saniculae
COL308. Hylaeus annulatus
Missing lines indicate failed sequencing reaction or unavailable material

## Slide 2
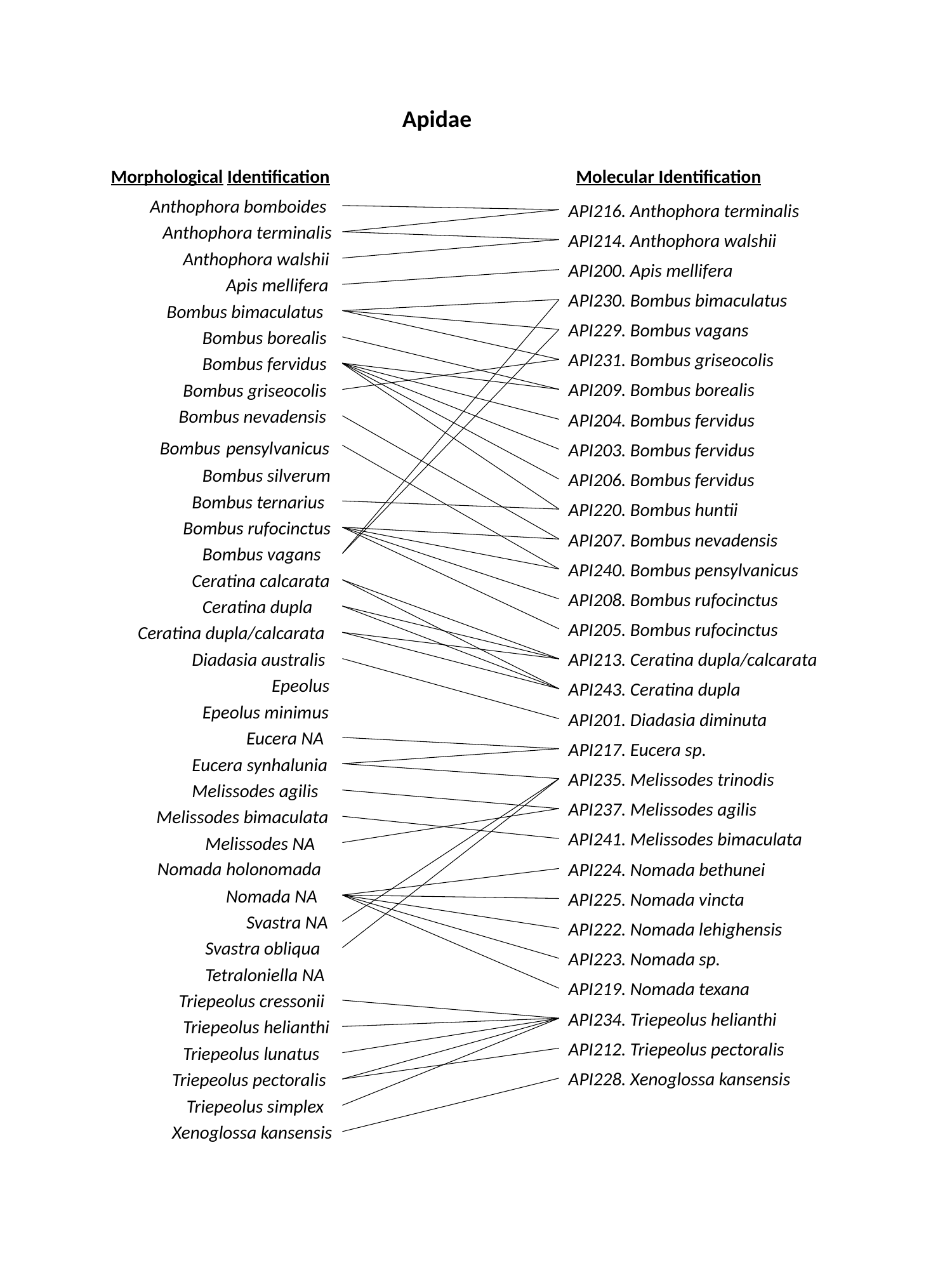

Apidae
Morphological Identification
Molecular Identification
Anthophora bomboides
API216. Anthophora terminalis
Anthophora terminalis
API214. Anthophora walshii
Anthophora walshii
API200. Apis mellifera
Apis mellifera
API230. Bombus bimaculatus
Bombus bimaculatus
API229. Bombus vagans
Bombus borealis
API231. Bombus griseocolis
Bombus fervidus
API209. Bombus borealis
Bombus griseocolis
Bombus nevadensis
API204. Bombus fervidus
Bombus pensylvanicus
API203. Bombus fervidus
Bombus silverum
API206. Bombus fervidus
Bombus ternarius
API220. Bombus huntii
Bombus rufocinctus
API207. Bombus nevadensis
Bombus vagans
API240. Bombus pensylvanicus
Ceratina calcarata
API208. Bombus rufocinctus
Ceratina dupla
API205. Bombus rufocinctus
Ceratina dupla/calcarata
Diadasia australis
API213. Ceratina dupla/calcarata
Epeolus
API243. Ceratina dupla
Epeolus minimus
API201. Diadasia diminuta
Eucera NA
API217. Eucera sp.
Eucera synhalunia
API235. Melissodes trinodis
Melissodes agilis
API237. Melissodes agilis
Melissodes bimaculata
API241. Melissodes bimaculata
Melissodes NA
Nomada holonomada
API224. Nomada bethunei
Nomada NA
API225. Nomada vincta
Svastra NA
API222. Nomada lehighensis
Svastra obliqua
API223. Nomada sp.
Tetraloniella NA
API219. Nomada texana
Triepeolus cressonii
API234. Triepeolus helianthi
Triepeolus helianthi
API212. Triepeolus pectoralis
Triepeolus lunatus
API228. Xenoglossa kansensis
Triepeolus pectoralis
Triepeolus simplex
Xenoglossa kansensis

## Slide 3
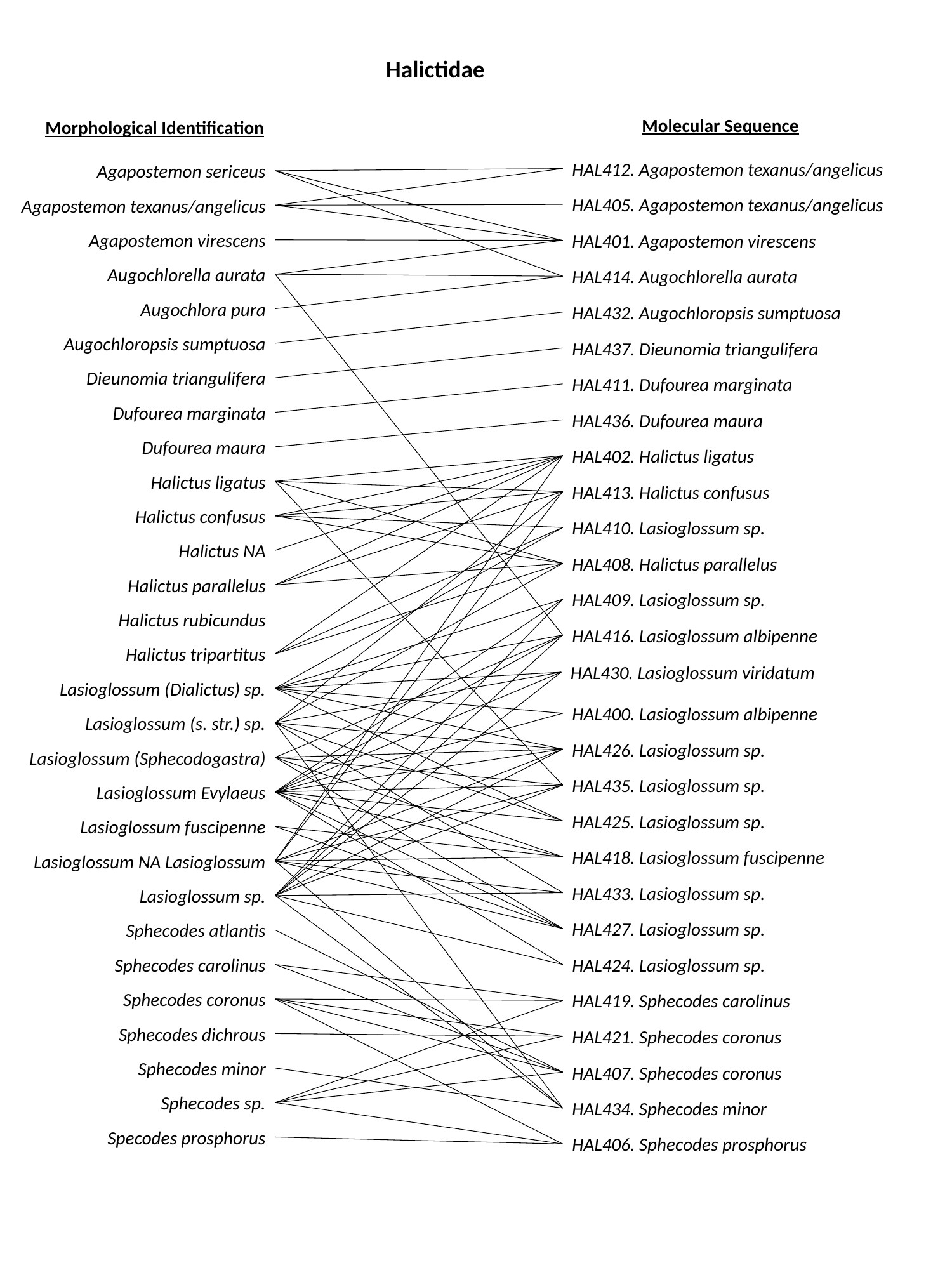

Halictidae
Molecular Sequence
Morphological Identification
HAL412. Agapostemon texanus/angelicus
Agapostemon sericeus
HAL405. Agapostemon texanus/angelicus
Agapostemon texanus/angelicus
Agapostemon virescens
HAL401. Agapostemon virescens
Augochlorella aurata
HAL414. Augochlorella aurata
Augochlora pura
HAL432. Augochloropsis sumptuosa
Augochloropsis sumptuosa
HAL437. Dieunomia triangulifera
Dieunomia triangulifera
HAL411. Dufourea marginata
Dufourea marginata
HAL436. Dufourea maura
Dufourea maura
HAL402. Halictus ligatus
Halictus ligatus
HAL413. Halictus confusus
Halictus confusus
HAL410. Lasioglossum sp.
Halictus NA
HAL408. Halictus parallelus
Halictus parallelus
HAL409. Lasioglossum sp.
Halictus rubicundus
HAL416. Lasioglossum albipenne
Halictus tripartitus
HAL430. Lasioglossum viridatum
Lasioglossum (Dialictus) sp.
HAL400. Lasioglossum albipenne
Lasioglossum (s. str.) sp.
HAL426. Lasioglossum sp.
Lasioglossum (Sphecodogastra)
HAL435. Lasioglossum sp.
Lasioglossum Evylaeus
HAL425. Lasioglossum sp.
Lasioglossum fuscipenne
HAL418. Lasioglossum fuscipenne
Lasioglossum NA Lasioglossum
HAL433. Lasioglossum sp.
Lasioglossum sp.
HAL427. Lasioglossum sp.
Sphecodes atlantis
Sphecodes carolinus
HAL424. Lasioglossum sp.
Sphecodes coronus
HAL419. Sphecodes carolinus
Sphecodes dichrous
HAL421. Sphecodes coronus
Sphecodes minor
HAL407. Sphecodes coronus
Sphecodes sp.
HAL434. Sphecodes minor
Specodes prosphorus
HAL406. Sphecodes prosphorus

## Slide 4
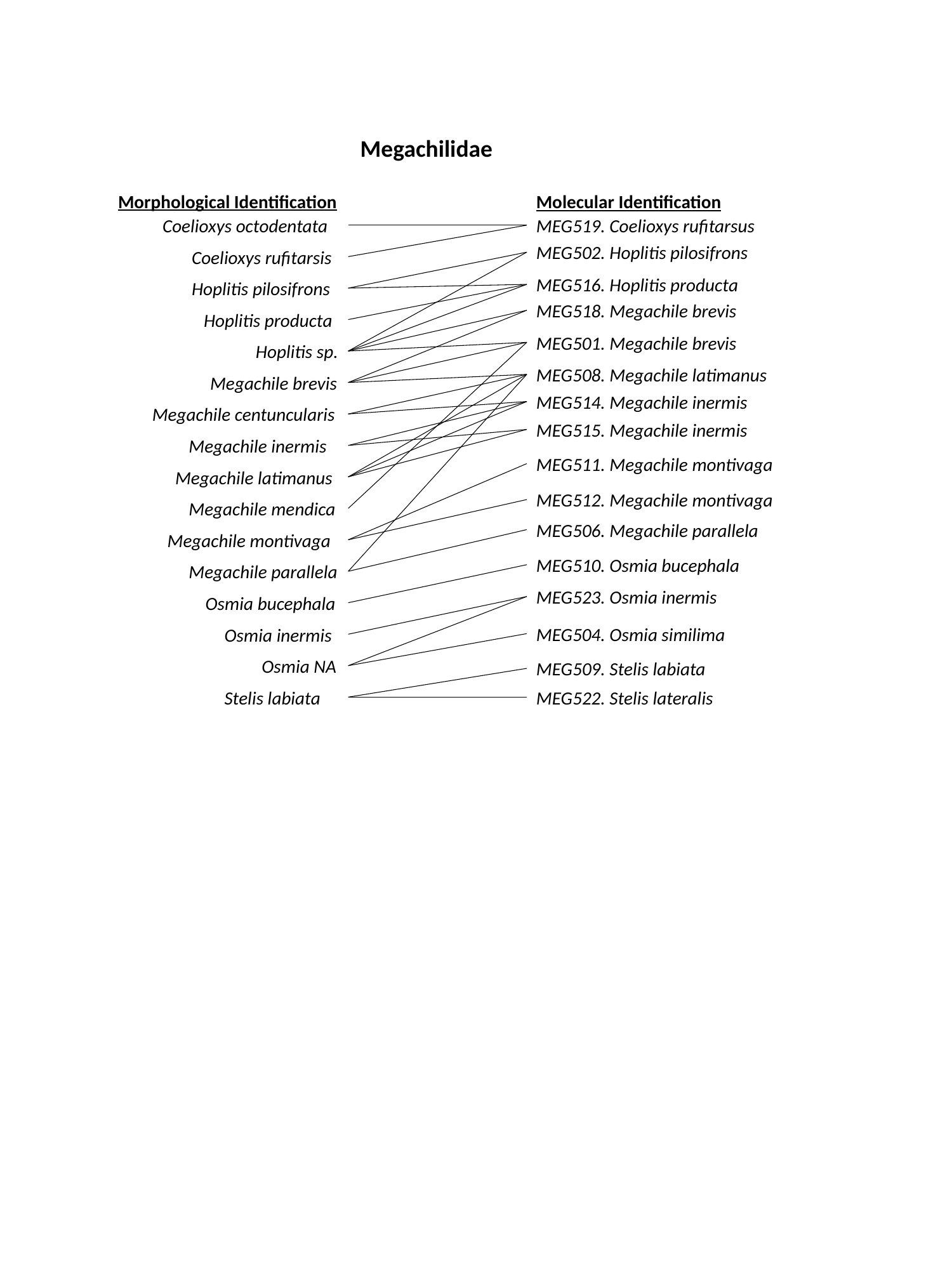

Megachilidae
Morphological Identification
Molecular Identification
Coelioxys octodentata
MEG519. Coelioxys rufitarsus
MEG502. Hoplitis pilosifrons
Coelioxys rufitarsis
MEG516. Hoplitis producta
Hoplitis pilosifrons
MEG518. Megachile brevis
Hoplitis producta
MEG501. Megachile brevis
Hoplitis sp.
MEG508. Megachile latimanus
Megachile brevis
MEG514. Megachile inermis
Megachile centuncularis
MEG515. Megachile inermis
Megachile inermis
MEG511. Megachile montivaga
Megachile latimanus
MEG512. Megachile montivaga
Megachile mendica
MEG506. Megachile parallela
Megachile montivaga
MEG510. Osmia bucephala
Megachile parallela
MEG523. Osmia inermis
Osmia bucephala
MEG504. Osmia similima
Osmia inermis
Osmia NA
MEG509. Stelis labiata
Stelis labiata
MEG522. Stelis lateralis
